# Effects and Mechanisms of Silibinin on Influenza A/H1N1 Pathogenesis in a Mouse Model

**DOI:** 10.1155/jotm/6618423

**Published:** 2025-01-16

**Authors:** Mohsen Keshavarz, Mohsen Ghorbani, Forough Shamsizadeh, Haideh Namdari, Vahid Salimi, Farhad Rezaei

**Affiliations:** ^1^The Persian Gulf Tropical Medicine Research Center, The Persian Gulf Biomedical Sciences Research Institute, Bushehr University of Medical Sciences, Bushehr, Iran; ^2^Department of Virology, School of Public Health, Tehran University of Medical Sciences, Tehran, Iran; ^3^Department of Parasitology and Mycology, School of Paramedicine, Bushehr University of Medical Sciences, Bushehr, Iran; ^4^Iranian Tissue Bank and Research Center, Tehran University of Medical Sciences, Tehran, Iran; ^5^School of Public Health, National Influenza Center, Tehran University of Medical Sciences, Tehran, Iran

**Keywords:** immunomodulatory effect, influenza A virus (IAV), lung damage, silibinin, silymarin

## Abstract

Silymarin is a polyphenolic flavonoid extracted from milk thistle. It has potent immunomodulatory effects and can inhibit the replication of influenza A virus (IAV). The present study aimed to determine the inflammatory and anti-inflammatory cytokine secretion patterns in mice before and after silibinin treatment. For this, bronchoalveolar lavage (BAL) fluids were collected from the thoracic cavity 5 days after the intervention, and viral quantification was performed using TaqMan Real-time PCR. Enzyme-linked immunosorbent assay (ELISA) was used to evaluate IFN‐γ and IL-10 levels in serum and BAL samples. Finally, pathological damage to lung tissue was assessed by pathologists. The results reveal that silibinin pretreatment exhibits a dose-dependent immunomodulatory effect on IFN-γ and IL-10 levels. After the virus challenge, silibinin reduced immune cell infiltration in mouse BAL fluid. These data similarly suggest a remarkable immunomodulatory effect of silibinin. Silibinin also decreased lung damage following the virus challenge in the post-treatment group, but its lung protective properties seem to be due to a different mechanism than when it was administered before infection. Finally, high doses of silibinin (post-treatment) significantly reduced viral load in BAL fluid compared to the virus challenge group. These results support the idea that therapies aimed at moderating immune and inflammatory responses are essential to decrease the mortality rate caused by IAV infection. Silibinin has strong immunomodulatory properties, can inhibit IAV infection, and reduces lung tissue damage in a dose-dependent manner.

## 1. Introduction

Silymarin is a polyphenolic flavonoid extracted from milk thistle (*Silybum marianum*), a mixture containing four isomeric flavonolignans, including silibinin, silydianin, silybin, and silychristin [1]. Among these contents, silibinin has great importance due to its wide range of performance, such as anti-cancer, antioxidant, anti-inflammatory, and antiviral properties [2, 3].

Studies have shown silymarin, and its derivatives possess intense antiviral activities against the various viral families by targeting multiple steps of the viral replication [4]. This makes silymarin and its components, particularly silibinin, promising candidates for therapeutic development against various viral infections.

Influenza A virus (IAV) is a highly contagious respiratory virus that poses a serious threat to both human and animal populations. It has been estimated this virus causes over 250,000 death tolls worldwide annually [5, 6]. Although effective vaccines are available in the market against IAV as prophylaxis, due to frequent antigenic drift and shift during replication, regularly updates are required [7]. Moreover, due to few IAV antiviral therapies and the emerging resistance strain of IAV, identifying novel strategies to expand effective treatments against this contagious pathogen is highly important [8]. In this context, natural compounds with antiviral and immunomodulatory properties [9, 10], such as silibinin, offer an attractive alternative or adjunctive strategy.

Previous studies have demonstrated that silymarin exhibits antiviral activity against IAV by inhibiting viral replication in a dose-dependent manner, primarily by disrupting late-stage mRNA synthesis in vitro [11]. However, the precise mechanisms by which silibinin and its derivatives affect different stages of the influenza virus life cycle remain incompletely understood, and more detailed investigations are required to elucidate these antiviral effects.

On the other side, with its derivatives, silymarin has potent immunomodulatory effects and elicits anti-inflammatory responses via suppression of a wide range of cytokines such as IFN-γ and IL-10 production [12, 13]. Moreover, suppression of MAPK/ERK1/2 signaling and release of IL-2 from T helper1 (Th1) control the organ damage caused by cytokine storm throughout the acute phase of influenza infection [14]. Recent studies have shown that silibinin pretreatment could protect mice against lung injury through the production of specific proinflammatory cytokines as well as LPS-induced recruitment of inflammatory cells (neutrophils, macrophages, and T-cells) into airway epithelium [15]. This dual role both modulating inflammation and facilitating immune cell recruitment, suggests that silibinin may help balance the immune response to viral infections, mitigating tissue damage while maintaining pathogen clearance.

Given that an excessive immune response is a key factor in the morbidity and mortality associated with influenza infections, particularly through cytokine storms, therapies that can moderate the immune and inflammatory response without completely suppressing antiviral defenses are critically important [16, 17]. Therefore, further investigation into the specific cytokine expression profiles induced by silibinin treatment before and after IAV infection is necessary to understand its therapeutic potential better.

Since the most common cause of death in influenza patients is intensive responses of immunity, using of therapies to moderate immune and inflammatory responses is critical in reducing the mortality rate. The present study aimed to determine the inflammatory and anti-inflammatory cytokines expression pattern following the administration of silibinin in mice before and after influenza infection.

## 2. Materials and Methods

### 2.1. Cell Culture and Influenza Virus Propagation

MDCK cells were obtained from Tehran University of Medical Sciences (TUMS), Department of virology, and were cultivated in Dulbecco's Modified Eagle's Medium (DMEM) supplemented with 10% Fetal Bovine Serum (FBS) (Gibco USA) and 2 mM Pen/Strep (Gibco USA) at 37°C in a 5% CO_2_ incubator. The influenza virus, A/Puerto Rico/8/1934 (H1N1) strain, was obtained from the Department of Virology, TUMS, and was grown on MDCK-SIAT1. As a medium, we used serum-free DMEM supplemented with 1 μg/mL of Trypsin-TPCK (Sigma, USA). RT-PCR on matrix gene was used to confirm influenza virus replication, and virus infectivity dose was measured using (TCID50) in combination with the hemagglutination assay (HA).

### 2.2. Animal Model and Experimental Design

Female 7–8-week-old BALB/C mice (weight, 18–20 g) were purchased from Pasteur Institute of Iran (Alborz-Iran). Before starting the study, mice were quarantined for 1 week at 25°C with water and food under a 12 h day-night cycle. All experiment procedures were approved according to the Ethical Committee of TUMS (ethics number: IR.TUMS.SPH.REC. 1397.159).

We divided our study into 2 main experiments. In each experiment, there were 4 mice groups, each group containing 6 mice, including PBS control, virus challenge, and 2 other groups that received silibinin in two different doses (25 and 50 mg/kg). In the pretreatment (first experiment), mice groups received silibinin for 7 days (25 and 50 mg/kg, Sigma-Aldrich) through the intraperitoneal route before the virus challenge. After that, mice groups challenge with the 5LD50 influenza virus (H1N1/PR8) intranasally. In the post-treatment (second experiment), the mice group was challenged with the 5LD50 influenza virus (H1N1/PR8) intranasally. After that received silibinin for 7 days (25 and 50 mg/kg, Sigma-Aldrich) through the intraperitoneal route. All experimental groups were monitored for weight and clinical signs, during this period.

### 2.3. Bronchoalveolar Lavage (BAL) Cells' Counting and Viral Load

Three mice were euthanized in each group (5 days after the last intervention), and BAL fluids were collected from the thoracic cavity by rinsing the lung with 1 mL of cold phosphate-buffered saline (PBS). The BAL samples in each group were centrifuged (2500 RPM for 10 min), pellet cells were stained with Giemsa dye, and the supernatants were stored at −80°C for viral quantification using TaqMan Real-time PCR assay. For the evaluation of leukocyte percentage in BAL fluid, we used an optical microscope. Each slide was counted three times, and the lymphocyte, monocyte, and neutrophil percentages were calculated based on the morphological features.

As mentioned above, evaluation of viral load in BAL fluid was performed by TaqMan Real-time PCR. The BAL fluid supernatant was subjected to viral RNA extraction kit (Roche Diagnostics, Germany) according to the manufacture procedure. Subsequently, the viral load assay was carried out using the one-step TaqMan real-time PCR master mix (ADD bio, Korea) and specific primers and probes (Table 1). The temperature protocol was as follows: 43°C/30 min (reverse transcription) and 94°C/10 min (polymerase activation), followed by 50 cycles at 94°C/15 s (Denaturation) and 60°C/60 s (Annealing and Extension).

### 2.4. ELISA Assay

Five days after the last intervention, three mice per group were sacrificed, and both serum and BAL fluid samples were collected. Cytokine levels of IL-10 and IFN-γ were measured using enzyme-linked immunosorbent assay (ELISA). For serum and BAL samples, ELISA kits specific for IFN-γ (Invitrogen) and IL-10 (R&D Systems, Minneapolis, MN) were used according to the manufacturer's instructions.

In brief, 100 μL of capture antibody was added to each well of the ELISA plates and incubated overnight at 4°C. Following incubation, the plates were treated with 200 μL of blocking buffer for 1 h and then washed three times with washing buffer. Next, 100 μL of double-diluted serum samples and BAL fluid were added to the wells. Polyclonal goat anti-mouse IgG conjugated with horseradish peroxidase (Avidin-HRP) was used as the secondary antibody. The reaction was developed using TMB (3,3′,5,5′-Tetramethylbenzidine) as the substrate, and the optical density was measured at 450 nm.

### 2.5. Histological Evaluation

Another characteristic that we evaluated in our experiment was the extent of pathological lung damage that may occur following influenza infection. Three mice in each group were sacrificed, and lung tissue was discarded and fixed in 10% (v/v) formalin solution and paraffin-embedded. Finally, the slides were stained with hematoxylin and eosin (H&E) stains. To evaluate and compare the pathological damage to the lungs, the slides were examined under a light microscope and based on normal and abnormal peribronchial and perivascular counts; each slide was assigned a pathology score. The slides were reviewed independently by three independent pathologists.

### 2.6. Statistical Analysis

All data analysis was performed by GraphPad Prism software, version 8. Multiple comparisons were performed by one-way ANOVA test. *p* value < 0.05 was considered statistically significant.

## 3. Results

### 3.1. Effects of Silibinin on Mice Weight Before and After Virus Challenging

In the pretreatment group, silibinin solution in two doses (25 and 50 mg/kg) was injected through the intraperitoneal route for 7 days. After that, the mice groups were challenged by the 5LD50 H1N1 influenza virus. In contrast, in the post-treatment, mice groups were challenged by influenza, and then silibinin solution was injected intraperitoneally for 7 days. All the mice groups were followed for 5 days and then evaluated for the outcome.

**Figure 1:**
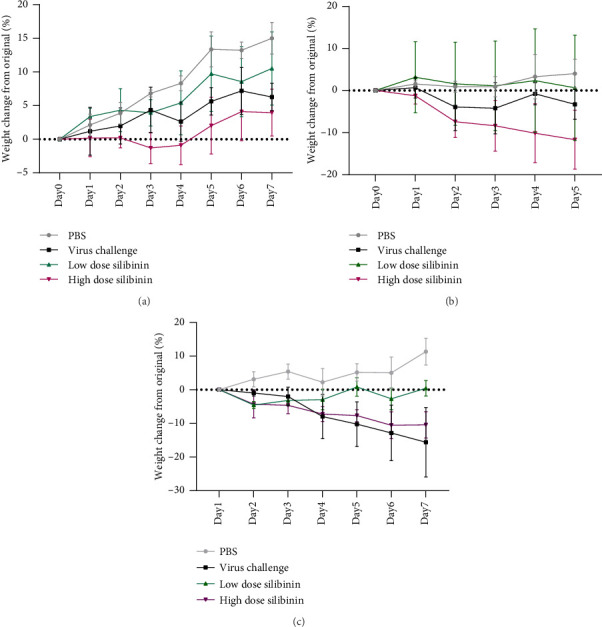
Effects of silibinin on mice weight. After 7 days of only silibinin administration, there were no significant differences between the silibinin groups and the control group (a). A high-dose silibinin group significantly reduced mice weight after the H1N1 influenza challenge (first experiment) (b). Additionally, in the second experiment, the high-dose silibinin group significantly reduced mice's weight following the virus challenge (c).

Weight loss is one of the primary pieces of evidence that could evaluate the severity of illness. Although we saw a bit of weight loss in the high dose of the silibinin group, there was no significant difference between other groups prior to the virus challenge (Figure 1(a)). In the pretreatment, our results showed a significant weight loss between the high doses of the silibinin group compared to the PBS control (*p* value = 0.0391). However, no significant difference was observed between low doses of the silibinin group compared to the PBS control (Figure 1(b)). Also, the results of the second experiment showed a significantly reduced weight loss in the high dose group compared to the PBS control (*p* value = 0.0005). However, no significant weight loss was observed between the low dose and the PBS group (Figure 1(c)).

### 3.2. Silibinin Pretreatment Induced Inflammatory and Anti-Inflammatory Responses Before Virus Challenge

**Figure 2:**
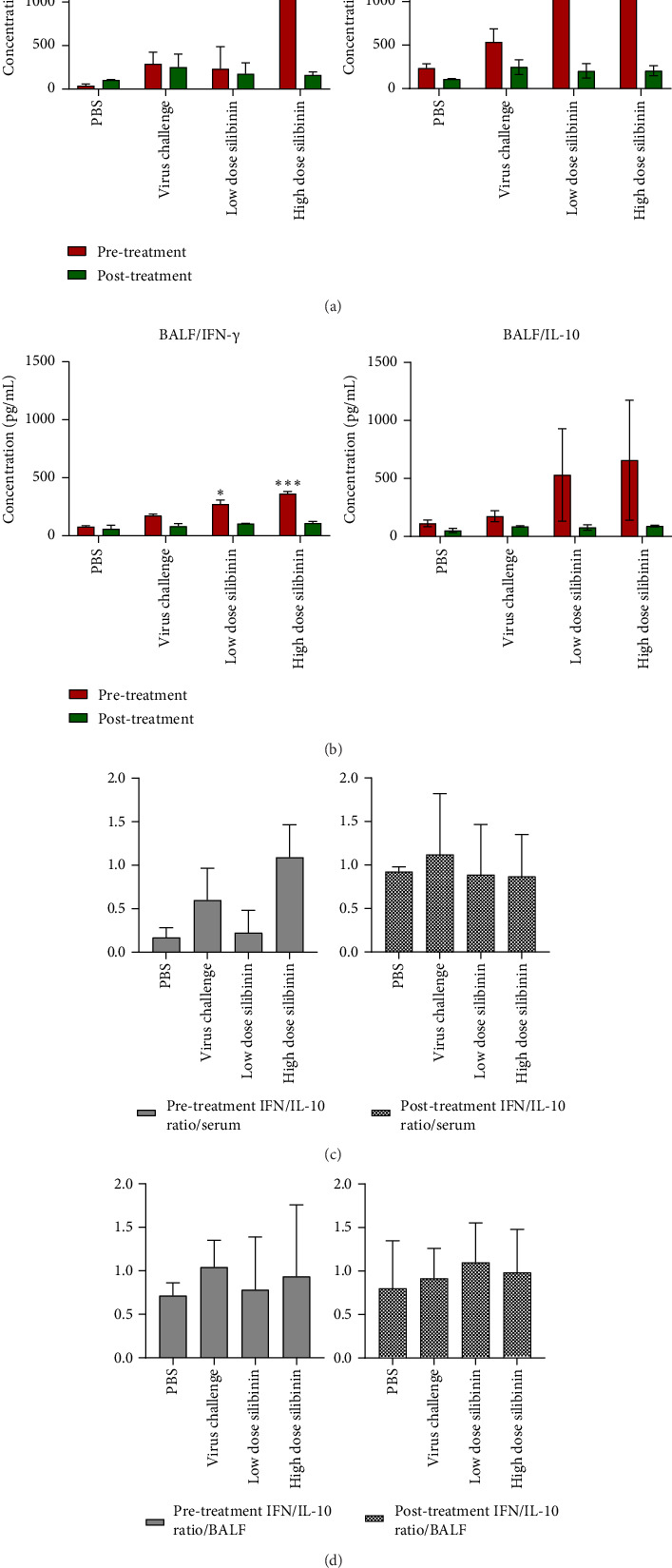
Silibinin induced inflammatory and anti-inflammatory responses. Pretreatment with high doses of silibinin significantly increased IFN production (a). In addition, IL-10 levels were significantly elevated following silibinin pretreatment (a). In contrast, upon challenge with the influenza virus (post-treatment), no significant differences were observed in IFN and IL-10 cytokines (a). Pretreatment with silibinin increased IFN-γ production in BAL samples in a dose-dependent manner, with significant increases at 25 and 50 mg doses (b). IL-10 levels in BAL samples also increased in both dose groups, but this was not statistically significant (b). In contrast, after the post-treatment, no significant differences in IFN-γ or IL-10 levels were observed in BAL samples between groups (b). Also, graphs represent the ratio of IFN-γ/IL-10 in serum (c) and bronchoalveolar lavage fluid (BALF) (d) of pretreated and post-treated animals. ⁣^∗^*p*  <  0.05; ⁣^∗∗∗^*p*  <  0.001.

Given the critical role of immune response intensity in infection inhibition and tissue damage prevention, we examined the role of two key cytokines and evaluated the immune-modulatory effects of silibinin. In the pretreatment, the level of IFN-γ in the high-dose silibinin group was significantly higher compared to the PBS control group (*p*=0.01), whereas no significant difference was observed in the low-dose group (*p* value > 0.05) (Figure 2(a)). Additionally, the level of IL-10 significantly increased in both silibinin-treated groups compared to the PBS control group (*p* value < 0.05) (Figure 2(a)).

In the post-treatment, the levels of IL-10 were not significantly different between the groups. Although no significant differences in IFN-γ levels were observed in these groups, we noted that the reduction in IFN-γ was dose-dependent. Our data indicate that silibinin pretreatment exhibits a dose-dependent anti-inflammatory effect, particularly on IFN-γ (Figure 2(a)).

In addition to the serum analysis, we further evaluated the levels of IFN-γ and IL-10 in BAL fluid. The results showed that IFN-γ levels in the pretreatment group were dose-dependent, with a significant increase in the low dose (25 mg) silibinin group (*p* < 0.05) and a more pronounced increase in the high dose (50 mg) silibinin group (*p* value < 0.001) (Figure 2(b)). However, while IL-10 levels increased in both silibinin-treated groups compared to the PBS control, this increase was not statistically significant (*p* value > 0.05) (Figure 2(b)).

In the post-treatment groups, there were no significant differences in the levels of either IFN-γ or IL-10 between the silibinin-treated groups and the PBS control group (*p* value > 0.05) (Figure 2(b)).

Figures 2(c) and 2(d) present the ratio of IFN-γ to IL-10 in pretreated and post-treated animals, demonstrating how the balance between these two cytokines might influence lung damage.

### 3.3. Silibinin Reduced Immune Cells Infiltration in Mouse BAL Fluid

It is obvious that cellular immunity has a critical role in viral clearance, but the severe immune response and cell infiltration abundance could be harmful effects. To assess immune cell distribution in the lung after administration of silibinin, the BAL fluid was collected from the pulmonary cavity of each mice group (3 mice per group) by washing the lungs with 1 mL of cold PBS.

**Figure 3:**
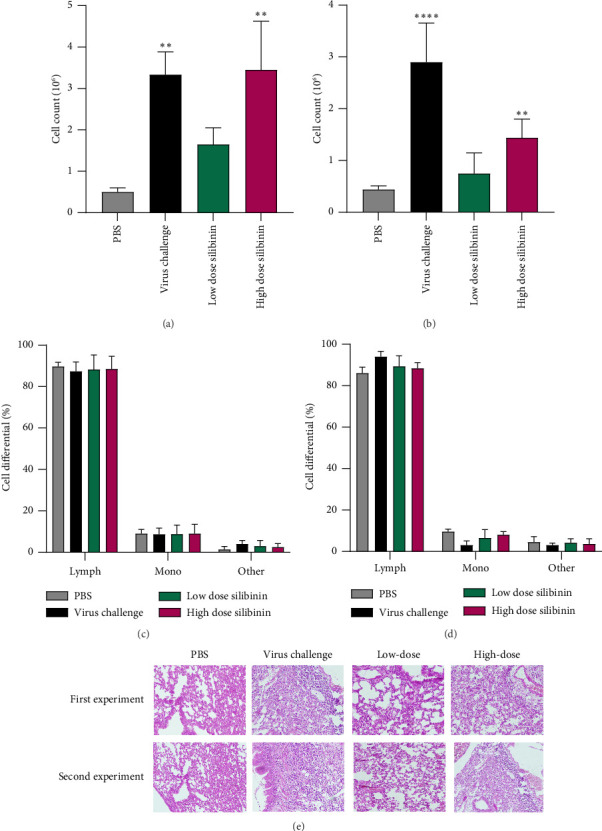
Silibinin reduced immune cells infiltration in BAL fluid. Low-doses silibinin pretreatment unlike high-doses and virus control groups significantly reduced immune cells infiltration in lung tissue (a). Low and high-doses silibinin post-treatment significantly reduced immune cells infiltration in lung tissue (b). Lymphocyte cells were the most abundant infiltrating cells in the lung tissue (c, d). (e) H&E staining showed distribution of immune cells in pre- and post-silibinin treatment. Bar = 100 μm.

Our result showed mouse treatment with a low dose of silibinin (the first experiment) significantly reduced immune cell distribution in BAL fluid compared to high dose and virus control group (*p* value = 0.03) (Figure 3(a)). Besides, the results of the post-treatment revealed the silibinin regime in both doses significantly reduced immune cell infiltration in BAL fluid compared to virus control (*p* value = 0.0001 and *p* value = 0.0012, respectively) (Figure 3(b)). In both experiments (Figures 3(c) and 3(d)), the lymphocyte cell was predominant in the BAL fluid of silibinin-treated groups. In addition, H&E staining in the pretreatment showed that high-dose silibinin group had a higher frequency of lymphocytes infiltrating than low-dose group (Figure 3(e)). In the post-treatment, silibinin high-dose and low-dose lung sections showed lower lymphocyte infiltration than the virus control group (Figure 3(e)).

### 3.4. Silibinin Decreased Lung Damage After Virus Infection

To assess the in vivo toxicity of silibinin in both experiments, various doses of silibinin were injected intraperitoneal and mice were followed for 5 days. None of the mice, including the mice injected with the high dose of silibinin (50 mg/kg), showed any visible abnormalities and were alive. To further investigate the effects of silibinin on lung tissue, pathological sections were examined in all mice groups.

**Figure 4:**
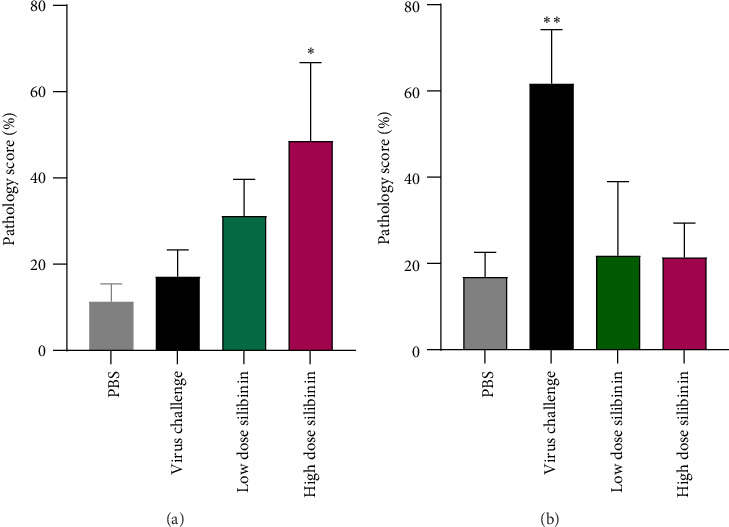
Silibinin attenuated lung damage after virus challenging. Lung damage was significantly induced by high-dose silibinin pretreatment (first experiment) (a). Post-treatment with silibinin (in both doses), however, significantly reduced lung damage (second experiment) (b). ⁣^∗^*p*  <  0.05; ⁣^∗∗^*p*  <  0.01.

Results of the pretreatment showed that silibinin in high dose significantly increased lung damage compared to the low dose of silibinin (*p* value = 0.02) and virus control (*p* value = 0.018), while there was no significant difference between low dose and virus challenge groups (Figure 4(a)). In addition, in the second experiment, in fact, the use of silibinin (in low and high doses) after the virus challenge significantly decreased lung tissue damage (*p* value = 0.002) (Figure 4(b)).

### 3.5. Silibinin in High Dose Reduced Viral Load in BAL Fluid

**Figure 5:**
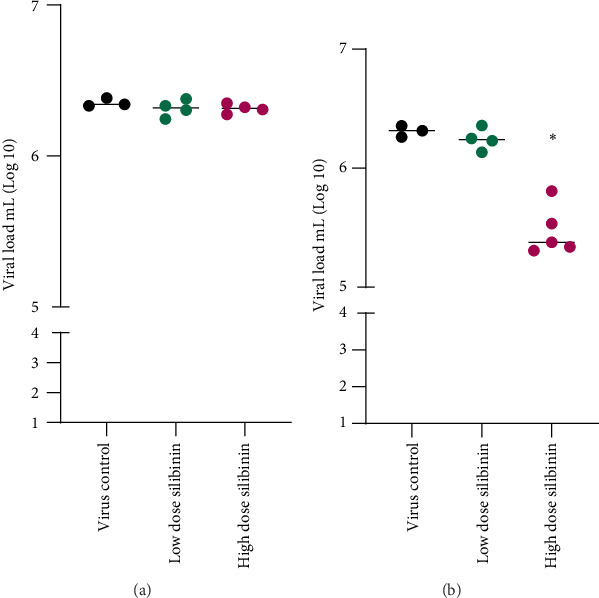
Silibinin reduced viral load in BAL fluid. Silibinin pretreatment did not have any effects on viral load (a). In contrast, post-treatment with high doses of silibinin significantly reduced the viral load in the BAL fluid (b)⁣^∗^*p* < 0.05.

Virological titers were evaluated in order to determine how silibinin affected viral replication. The first experiment showed silibinin pretreatment (in both doses) has no significant effect on viral load compared to the virus control group (Figure 5(a)). In contrast, the result of the second experiment showed that post-treatment with a high dose of silibinin significantly reduced viral load in BAL fluid compared to the virus control group (*p* value < 0.05), while the low dose of silibinin had no significant effect on reducing viral load (Figure 5(b)).

## 4. Discussion

Many nations have a rich history of using plants as medicine, and even today, medicinal plants continue to be widely used due to their affordability and accessibility [18, 19]. For thousands of years, nature has provided important medicines, and many modern drugs come from natural sources. Plants have been used in traditional remedies to treat various illnesses, and the different natural compounds they contain have helped create many of today's medicines. As scientists continue to explore the world's biodiversity, much of which is still undiscovered, natural products will remain essential for finding new drugs [20].

Infections with influenza cause respiratory symptoms as well as fever, chills, headache, muscle pain, fatigue, and cough. If it progresses, it causes complications such as bronchitis, pneumonia, secondary bacterial infections, and heart problems, which are more common in immunocompromised patients, the elderly, and children. According to estimates from the World Health Organization (WHO), influenza is responsible for 290,000 to 650,000 deaths annually from respiratory diseases alone, excluding fatalities related to other conditions that may be linked to influenza [21]. It also causes significant financial losses globally [22]. Because infection with this virus does not result in immunity to subsequent diseases, vaccines against it must be changed annually. Additionally, the emergence of drug-resistant strains of the virus is another critical concern that must be closely monitored during the use of anti-influenza drugs [23].

One of the therapeutic compounds derived from the thistle plant, known for its high medicinal properties, is silibinin [24]. This substance effectively treats a wide range of illnesses, including cancers, viral infections, and inflammatory diseases [25–27]. On this substance, numerous studies have been done with acceptable results.

This substance's anti-inflammatory and immunomodulatory properties are examples of its most crucial and significant properties [28]. Inflammation of the respiratory system is one of the primary and severe complications of influenza virus infection [29]. This substance can be an excellent choice to demonstrate the impact of plant derivatives on the modulation and regulation of the immune system in the inflammation caused by the disease.

In the pretreatment, silibinin was administered to the treated groups to observe its effects on the mice. No noticeable weight loss was observed in any of the groups, indicating that silibinin posed no toxicity risk to the mice. In the pretreatment, the low-dose group showed no significant weight loss, while the high-dose group experienced notable weight loss. In the post-treatment, the low-dose group had no weight loss, and although the high-dose group initially lost weight, they recovered and gained weight by the end of the experiment. Monitoring the weight of the mice is a key indicator of their health, and any weight loss must be investigated. A 2020 study on the effects of silibinin on mouse fat tissue showed no significant weight changes after silibinin administration, confirming its lack of toxicity, which aligns with the findings of our study [30].

In influenza infection, immunopathogenesis caused by inflammation is a significant issue in pathogenicity [31]. Since some cytokines, such as IL-10 [32] and IFN-γ [29], are considered factors for measuring inflammation, in this experiment, an attempt has been made to investigate the effect of silibinin on these cytokines.

In this study, we investigated the immunomodulatory effects of silibinin on key cytokines, IFN-γ and IL-10, in both serum and BAL fluid during influenza infection. Pretreatment with silibinin, particularly at higher doses, significantly increased IFN-γ production in both serum and BAL samples, indicating enhanced immune activation both systemically and locally. Although IL-10 levels were elevated in silibinin-treated groups compared to PBS controls, the increase in BAL samples was not statistically significant, suggesting silibinin's balanced role in modulating both proinflammatory and anti-inflammatory responses to prevent tissue damage. In this regard, Schumann et al. showed that elevated IL-10 levels contributed to the reduction of both TH1 and TH2 immune responses, suggesting its role in limiting inflammation [33]. In post-treatment groups, no significant changes in cytokine levels were observed after virus exposure, indicating that silibinin's immunomodulatory effects are more effective when used as a preventive measure rather than as a treatment once infection has already been established. These data emphasize silibinin's potential as a preventive intervention in modulating immune responses during influenza infection.

In one study, by investigating the protective effect of silibinin on liver damage caused by T cells in mice, they concluded that silibinin probably increases anti-inflammatory factors such as IL-10 and reduces a set of inflammatory factors by affecting liver Kupffer cells. It strongly prevents liver damage caused by T cells [33]. The results of our study are consistent with this study. Our results showed that although high doses of silibinin before infection increased lung damage, post-infection treatment significantly reduced lung tissue damage and accelerated recovery, highlighting its potential protective role in the lungs through immune modulation.

A study on rheumatoid arthritis showed that high-dose silibinin significantly reduced inflammatory markers (ESR, IL-8, IL-6, TNF-α, anti-CCP) and increased Hb, IL-10, and IL-2, compared to placebo [34]. Similarly, our study found that silibinin's immunomodulatory effects vary by dose and timing, with its anti-inflammatory properties likely reducing influenza complications by lowering inflammation.

A study on chronic HCV patients treated with silibinin and vitamin E showed anti-inflammatory effects by modulating cytokines like IFN-γ, TNF-α, IL-4, and IL-6 [35].

Our study found that IL-10 and IFN-γ levels increased in the high-dose group, with a more significant rise in IFN-γ. Although higher IL-10 levels were expected to reduce immune cell infiltration, inflammation and lung damage increased, likely due to elevated IFN-γ. This suggests that the increased presence of immune cells in lung tissue contributed to the observed lung damage [36].

Finally, the amount of viral load was checked. When silibinin was consumed before exposure to the virus (pretreatment), the amount of viral load did not change. Still, when this substance was consumed after the virus exposure, it was observed that the viral load decreased in a high dose of silibinin. In a study evaluating the antiviral effect of silibinin derivatives, it was concluded that silibinin derivatives decrease the virus titer in mice that received influenza virus. These results were the same in in-vivo and in-vitro environments [37]. Further, another study investigating the inhibitory effect of silibinin on HIV-1 infection found evidence that supports the antiviral mechanism of silibinin on HIV and leads to a decrease in virus titer [38]. The results of the mentioned studies were consistent only with the high-dose silibinin group (injection of silibinin after exposure to the virus) in our study. These results demonstrated that silibinin might have a post-entry inhibitory effect.

According to the obtained data, it can be said that lower doses gave a better response than higher doses to improve the disease. The dose-dependent manner of silibinin has been shown in many studies, which is similar to our results. For example, in a study, it was determined by measuring the apoptosis induction effect in the A431 human epidermoid carcinoma cell line by silibinin that it inhibited A431 cell growth, inducing mitochondrial damage and apoptosis at a high dose [39].

This study has several limitations that should be acknowledged. First, while the use of a BALB/c mouse model provided valuable insights, the findings may not be fully generalizable to humans due to species-specific differences in immune responses. Additionally, the study focused primarily on IFN-γ and IL-10 as cytokine markers, potentially overlooking other critical inflammatory mediators involved in influenza pathogenesis. Furthermore, the precise mechanisms underlying silibinin's immunomodulatory and antiviral actions remain unclear, warranting further investigation into the molecular pathways involved. It is also important to note that the antiviral effect of silibinin was observed only in the post-treatment group receiving the high dose (50 mg). In contrast, the pretreated groups, despite showing higher IFN-γ levels, did not exhibit a significant reduction in viral load. This discrepancy suggests that the antiviral effects of silibinin may not be solely dependent on immune modulation through IFN-γ and IL-10, highlighting the need for further studies to clarify the factors contributing to viral load reduction.

## 5. Conclusion

This study found that medicinal herbs and their compounds have different effects according to the time and dosage. They are very effective if used correctly and lead to treatment and improvement of the symptoms and complications of the disease, but improper use worsens the condition. Silibinin, as a plant compound, has many therapeutic properties. Its most important property in the treatment of influenza is reducing the inflammation caused by immunopathogenesis. According to the studies, it seems this substance can be used as a complementary or alternative medicine for the patient, relevant to the expert physician's opinion.

## Figures and Tables

**Table 1:** Nucleotide sequences of primers and probes used for real-time TaqMan PCR.

Oligo name	Sequence
Flu A forward	GAC CRA TCC TGT CAC CTC TGA C
Flu A reverse	AGG GCA TTY TGG ACA AAK CGT CTA
Probe	(FAM)-TGC AGT CCT CGC TCA CTG GGC ACG-(BHQ1)

## Data Availability

Data will be made available on request.
